# Prediction of the invasion depth of superficial squamous cell carcinoma based on microvessel morphology: magnifying endoscopic classification of the Japan Esophageal Society

**DOI:** 10.1007/s10388-016-0527-7

**Published:** 2016-04-06

**Authors:** Tsuneo Oyama, Haruhiro Inoue, Miwako Arima, Kumiko Momma, Tai Omori, Ryu Ishihara, Dai Hirasawa, Manabu Takeuchi, Akihisa Tomori, Kenichi Goda

**Affiliations:** 1 Department of Endoscopy, Saku Central Hospital Advanced Care Center, Nagano, Japan; 2 Digestive Disease Center, Showa University Koto Toyosu Hospital, Tokyo, Japan; 3 Department of Gastroenterology, Saitama Cancer Center, Saitama, Japan; 4 Department of Endoscopy, Tokyo Metropolitan Cancer and Infectious Disease Center Komagome Hospital, Tokyo, Japan; 5 Center for Diagnostic and Therapeutic Endoscopy, Keio University, Tokyo, Japan; 6 Department of Gastrointestinal Oncology, Osaka Medical Center for Cancer and Cardiovascular Diseases, Osaka, Japan; 7 Department of Gastroenterology, Sendai City Medical Center, Miyagi, Japan; 8 Department of Gastroenterology, Niigata University Medical and Dental Hospital, Niigata, Japan; 9 Department of Gastroenterology, Saku Central Hospital Advanced Care Center, Nagano, Japan; 10 Department of Endoscopy, The Jikei University School of Medicine, 3-25-8 Nishi-shimbashi, Minato-ku, Tokyo, 105-8461 Japan

**Keywords:** Magnifying endoscopy, Esophageal cancer, Squamous cell carcinoma, Invasion depth, Japan esophageal society classification

## Abstract

Predicting invasion depth of superficial esophageal squamous cell carcinoma is crucial in determining the precise indication for endoscopic resection because the rate of lymph node metastasis increases in proportion to the invasion depth of the carcinoma. Previous studies have shown a close relationship between microvascular patterns observed by Narrow Band Imaging magnifying endoscopy and invasion depth of the superficial carcinoma. Thus, the Japan Esophageal Society (JES) developed a simplified magnifying endoscopic classification for estimating invasion depth of superficial esophageal squamous cell carcinomas. We conducted a prospective study to evaluate the diagnostic values of type B vessels in the pretreatment estimation of invasion depth of superficial esophageal squamous cell carcinomas utilizing JES classification, the criteria of which are based on the degree of irregularity in the microvascular morphology. Type A microvessels corresponded to noncancerous lesions and lack severe irregularity; type B, to cancerous lesions, and exhibit severe irregularity. Type B vessels were subclassified into B1, B2, and B3, diagnostic criteria for T1a-EP or T1a-LPM, T1a-MM or T1b-SM1, and T1b-SM2 tumors, respectively. We enrolled 211 patients with superficial esophageal squamous cell carcinoma. The overall accuracy of type B microvessels in estimating tumor invasion depth was 90.5 %. We propose that the newly developed JES magnifying endoscopic classification is useful in estimating the invasion depth of superficial esophageal squamous cell carcinoma.

## Introduction

Endoscopic resection (ER) can offer patients a curative and low-invasive treatment for superficial squamous cell carcinomas (SESCCs) [1]. Predicting invasion depth of SESCCs is crucial for determining the precise indication for endoscopic resection (ER) because the lymph node metastasis rate increases in proportion to the invasion depth of the SESCC [1–3]. According to the Japanese guidelines for diagnosis and treatment of esophageal cancer, T1a-EP or T1a-LPM SCC is considered an absolute indication for ER; T1a-MM or T1b-SM1, a relative indication; and T1b-SM2, an investigative stage (functionally speaking, a contraindication) [4, 5]. Subclassification of invasion depth of SESCC, the rate of lymph node metastasis, and the precise indication of ER are listed in Table 1 based on previous studies [1–5].

**Table 1:** Relationships among subclassification of invasion depth of SESCC, the rate of lymph node metastasis, and the precise indication of ER

Tumor depth of superficial esophageal squamous cell carcinoma			Lymph node metastasis rate (%)	Indication of endoscopic resection
T1a, Tumor invades mucosa (M)	EP	Carcinoma in situ (Tis)	0–3.3	Absolute
	LPM	Tumor invades lamina propria mucosa (LPM)		
	MM	Tumor invades lamina muscularis mucosa (MM)	0–12.2	Relative
T1b, Tumor invades submucosa (SM)	SM1	Tumor invades the submucosa to a depth of 200 µm or less from the muscularis mucosa	8–26.5	
	SM2	Tumor invades the submucosa to a depth more than 200 µm	22–61 %	Investigative stage^a^

^a^A contraindication

Endoscopic prediction of the depth of invasion is essential in making decisions regarding the indication for ER in SESCCs. Although predicting the invasion depth of SESCC is possible by conventional endoscopy, magnifying endoscopy provides a more accurate prediction of the tumor depth, in particular for superficial and flat-type SESCCs [6]. Inoue et al. [7–9] and Arima et al. [10–12] proposed magnifying endoscopic classifications based upon microvascular morphology and reported their diagnostic utility in predicting the histological invasion depth of SESCC as well as in deciding precise indications for ER. However, there are concerns that the multiplicity of classifications involving complicated criteria might confuse general endoscopists.

Hence, the committee of the Japan Esophageal Society (JES) developed a simplified classification for the magnifying endoscopic diagnosis (characterization and predicting invasion depth) of SESCC [13, 14] based on the Inoue and Arima classifications [7–12]. Subsequently, we, the committee members, conducted a prospective multicenter study to evaluate the JES classification for predicting the invasion depth of SESCC.

## Methods

### The JES classification

Mainly focused on the abnormal microvessels, this magnifying endoscopic classification offers simplified criteria not only for characterization but also for prediction of the invasion depth of SESCC [14]. This is essential for developing a treatment strategy for SESCC, in particular the indication for ER. Therefore, in this classification, morphological types of microvessels are classified into two categories of non-cancerous [normal epithelium, inflammation, and low grade intraepithelial neoplasia (LGIN)] and cancerous [high grade intraepithelial neoplasia (HGIN) and invasive SCC] lesions. The cancerous types of microvessels corresponding to histology of HGIN or invasive SCC are subclassified into three groups based upon an indication for ER as follows: an absolute indication type (HGIN, T1a-EP or T1a-LPM), a relative indication type (T1a-MM or T1b-SM1), and a contraindication type (T1b-SM2).

Diagnostic criteria of the JES classification are based on the degree of microvascular irregularity in the target lesion observed by magnifying endoscopy. Intrapapillary capillary loops (IPCL) are a basic unit of microvasculature in the squamous mucosal surface. The microvascular irregularity is evaluated for the presence or absence of each of the following morphological factors: weaving (i.e., tortuosity), dilatation, irregular caliber, and different shape (i.e., various shapes) [7, 8].

Microvessels are classified as type A if they have three or fewer factors and type B if they have all four. Type B is then subclassified into B1, B2, and B3 based on the running pattern or degree of dilatation of severely irregular microvessels. The JES classification criteria are summarized in Table 2 and mentioned in detail as follows:

**Table 2:** Summary and schema of the criteria of the JES magnifying endoscopic classification

Type of vessels	Schema	Definitions		Invasion depth	Histology
A		Normal IPCL or abnormal microvessels without severe irregularity^a^		No invasion	Normal epithelium, inflammation, and LGIN
B1		Abnormal microvessels with severe irregularity or highly dilated abnormal vessels	Type B vessels with a loop-like formation^b^	T1a-EP or T1a-LPM	HGIN and invasive SCC
B2			Type B vessels without a loop-like formation	T1a-MM or T1b-SM1	
B3			Highly dilated vessels which calibers appear to be more than three times that of usual B2 vessels^c^	T1b-SM2 or deeper	

*EP* epithelium, *LPM* lamina propria mucosae, *MM* muscularis mucosae, *SM* submucosa, *LGIN* low grade intraepithelial neoplasia, *HGIN* high grade intraepithelial neoplasia, *SCC* squamous cell carcinoma

^a^The caliber of type A vessels is about 7–10 μm [15]

^b^The caliber of B1 vessels is around 20 μm [15]

^c^The caliber of B3 vessels is often larger than 60 μm [15]

**Fig. 1:**
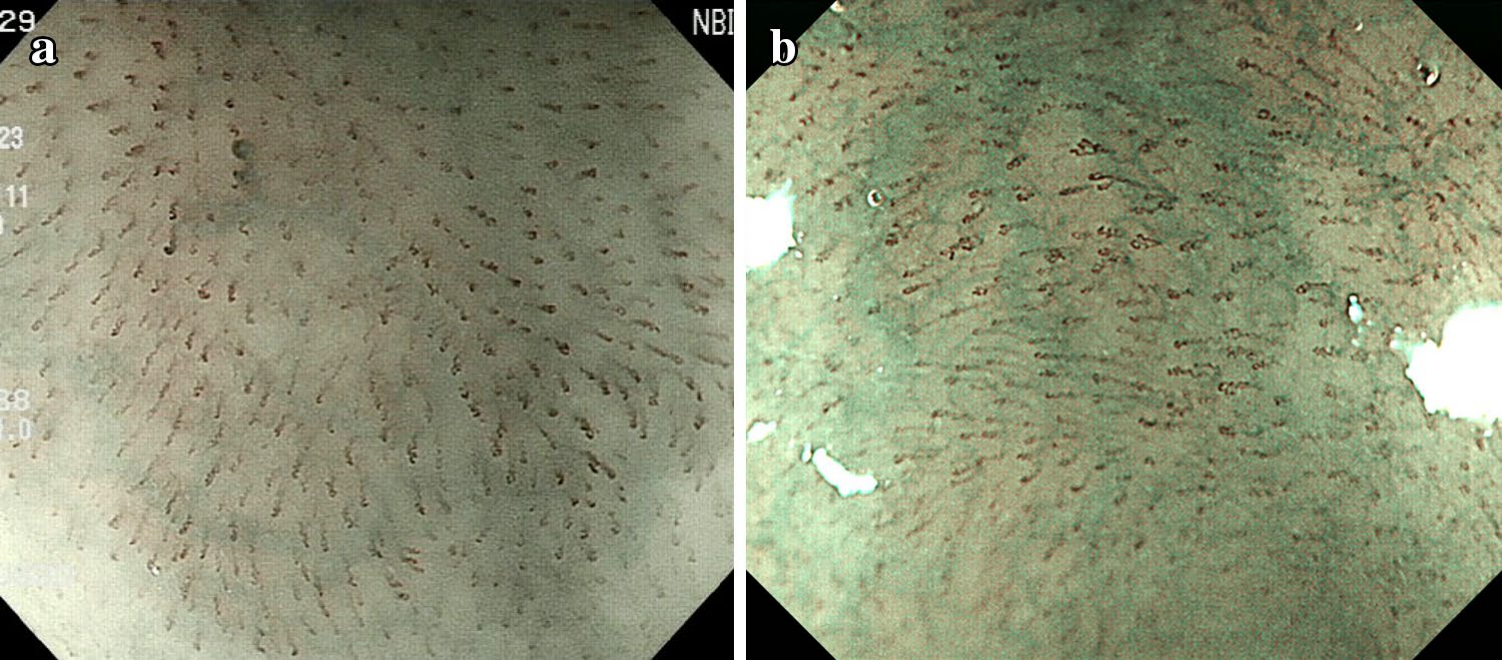
**a** Type A vessels of normal intrapapillary capillary loops. **b** Type A vessels of abnormal microvessels without severe irregularity

Type A: Normal IPCL (Fig. 1a) or abnormal microvessels without severe irregularity (Fig. 1b).

Type B: Abnormal microvessels with severe irregularity or highly dilated abnormal vessels.

**Fig. 2:**
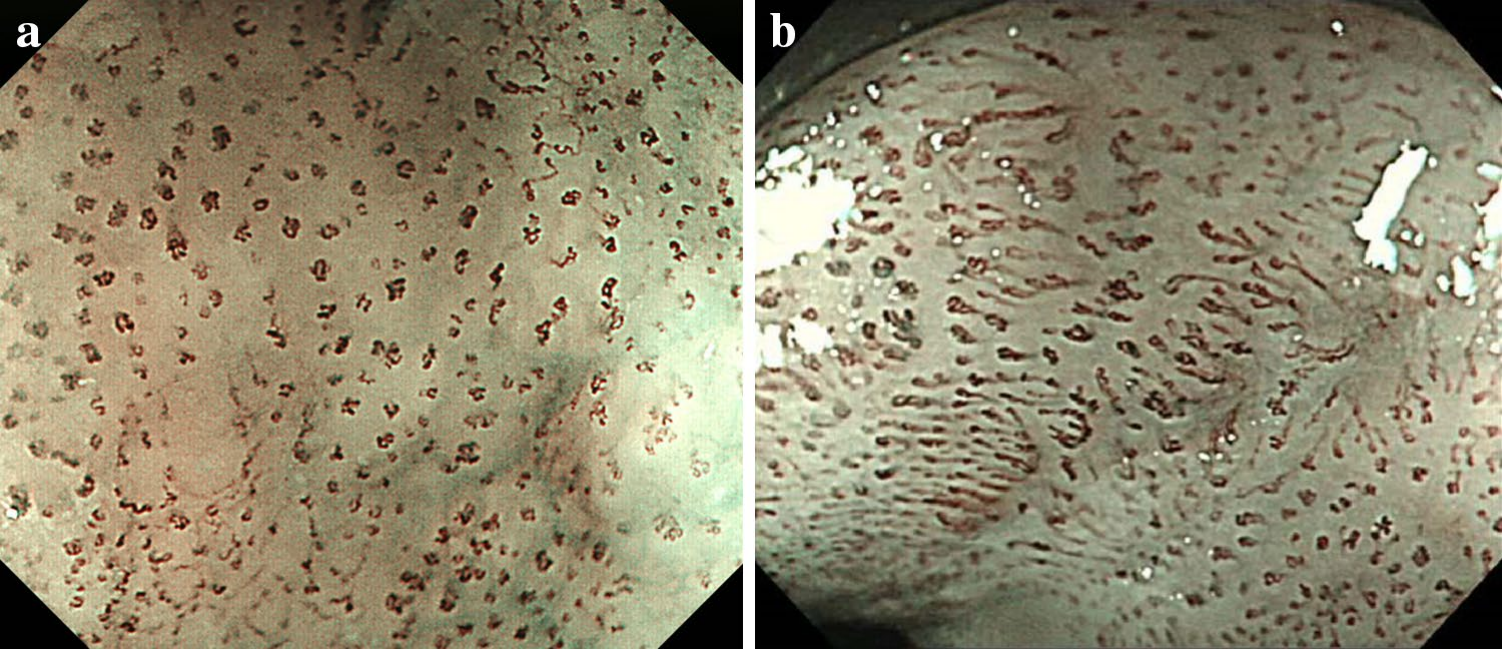
**a, b** Type B1 vessels with a loop-like formation

B1 is defined as type B vessels with a loop-like formation (Fig. 2a, b). The B1 vessels normally appear as dot-like microvessels in a target area (e.g., a brownish area) under NBI endoscopic observation with low or no magnification.

**Fig. 3:**
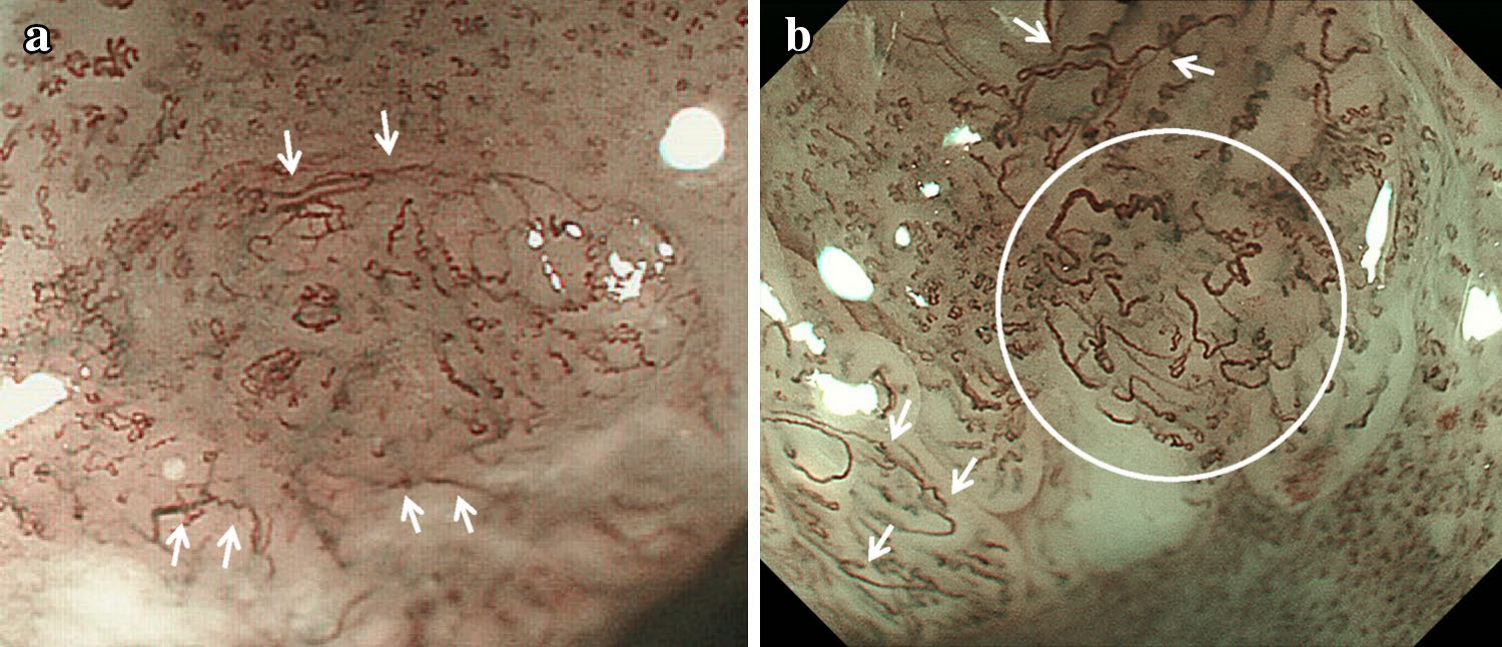
**a, b** Type B2 vessels without a loop-like formation (*white arrows* and inside a *white circle*)

B2 is defined as type B vessels without a loop-like formation that have a stretched and markedly elongated transformation. The B2 vessels often show a multilayered arrangement or an irregularly branched/running pattern (Fig. 3a, b).

**Fig. 4:**
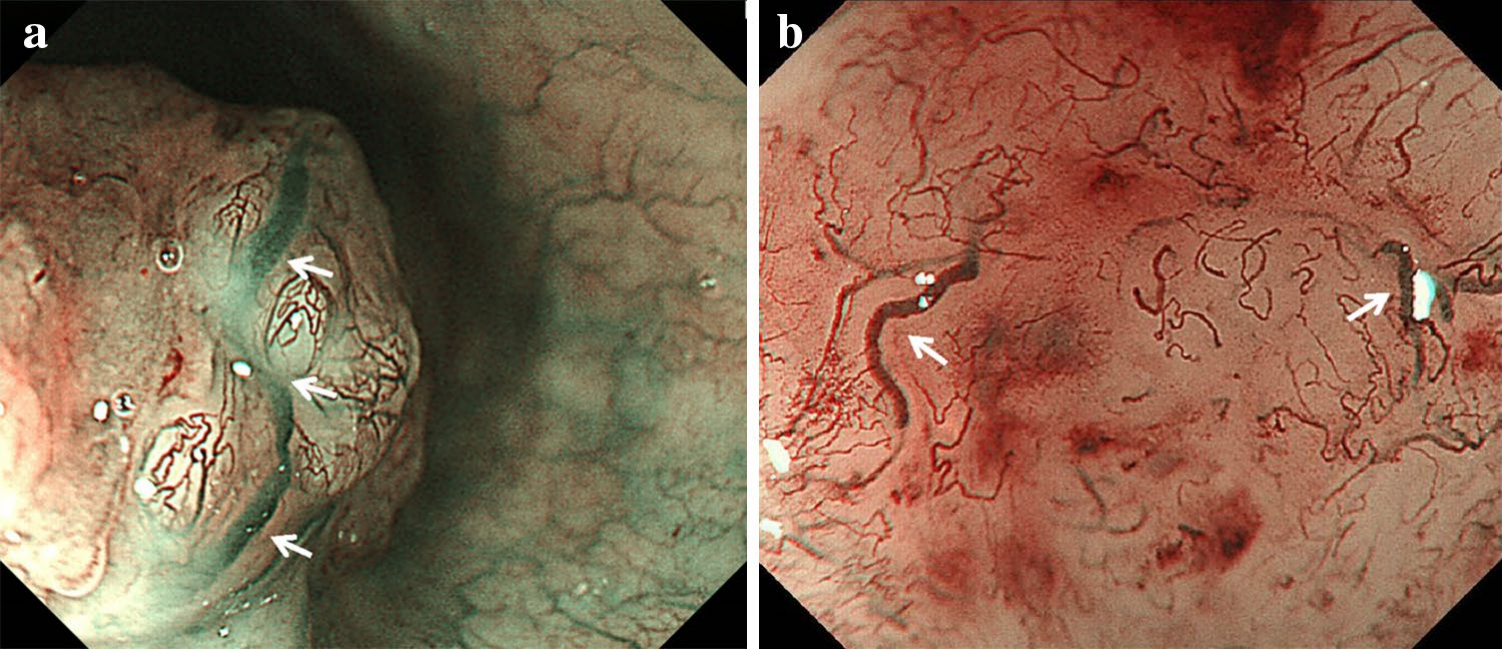
**a, b** Type B3 of highly dilated abnormal vessels (*white arrows*) whose caliber will be more than three times that of the B2 vessels around the B3 vessels

B3 is defined as highly dilated abnormal vessels whose caliber appears to be more than three times that of the usual B2 vessels and the B3 vessels often appear green in color (Fig. 4a, b) [15].

When target lesions have only type B1 vessels, the histological invasion depth is predicted as T1a-EP or T1a-LPM. When B2 and B3 vessels are seen in target lesions, the histological invasion depth is predicted as T1a-MM or T1b-SM1 and T1b-SM2 or deeper, respectively.

### Auxiliary criteria of the JES classification

#### Avascular area (AVA)

**Fig. 5:**
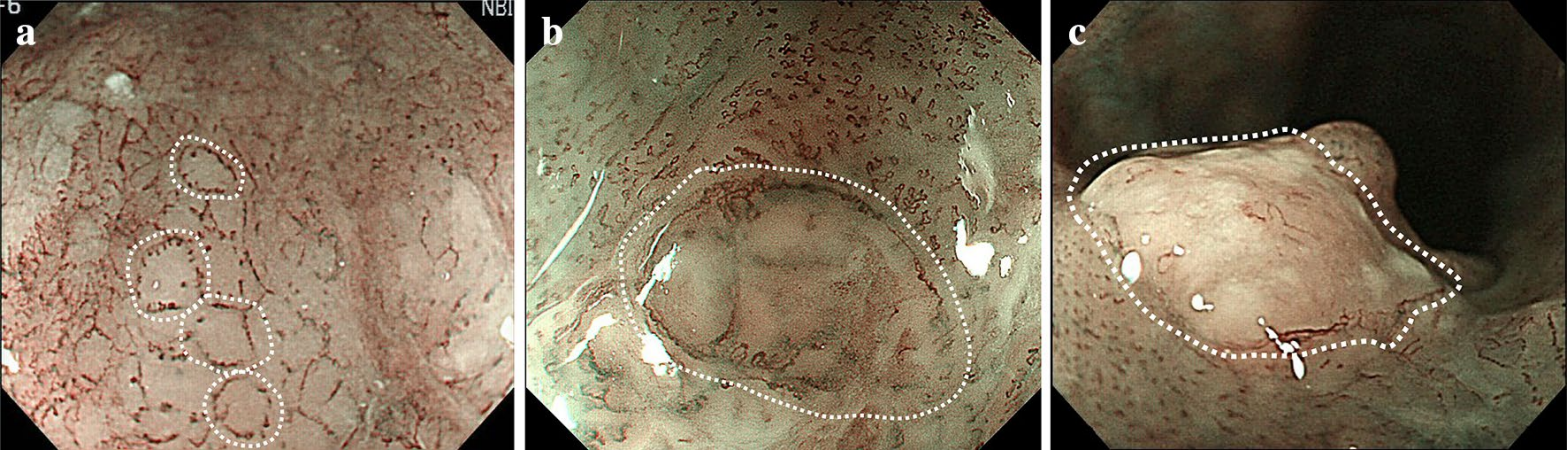
**a** Small-sized avascular area (AVA-small). **b** Middle-sized avascular area (AVA-middle). **c** Large-sized avascular area (AVA-large). Typical AVAs are shown by *white dotted lines*

**Fig. 6:**
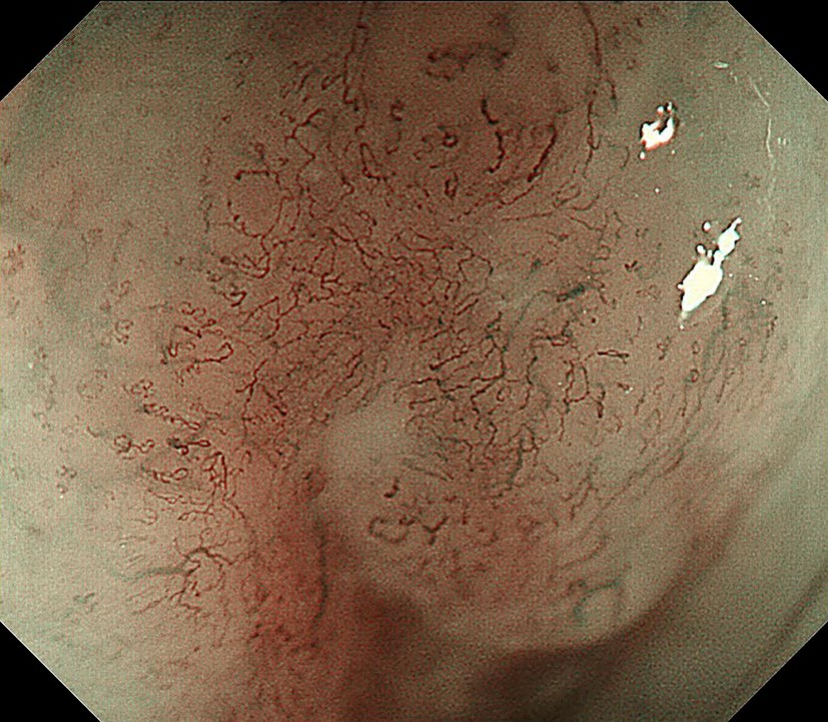
Reticular pattern vessels (Type R)

AVA was originally defined as a low or no vascularity area surrounded by stretched irregular vessels such as B2 or B3 vessels [11, 12]. The definition of AVA does not include the stretched irregular vessels in this classification. Thus, AVA is surrounded by all subtypes of type B microvessels including B1 vessels. [14]. Since a diameter of AVA is positively correlated with the histological invasion depth of SESCC, the AVA was categorized into three types as follows: AVA-small (smaller than 0.5 mm in diameter: Fig. 5), AVA-middle (0.5 mm or between 0.5 and 3 mm; Fig. 6), and AVA-large (3 mm or larger; Fig. 7) [11, 12]. Any types of AVA (small, middle, and large) surrounded by B1 vessels are suggestive of T1a-EP or T1a-LPM SCC. AVA-middle and AVA-large surrounded by B2 or B3 vessels are suggestive of T1a-MM or T1b-SM1 and T1b-SM2 invasive SCC, respectively [14].

#### Reticular pattern (Type R)

Reticular pattern vessels are defined as plexiform microvessels (Fig. 6) [11, 12]. This vascular pattern is often found in invasive SCC or non-SCC types of malignant epithelial neoplasms (e.g., basaloid (-squamous) carcinoma, adenosquamous carcinoma, and endocrine cell carcinoma) with an infiltrative growth pattern (i.e., INFc) composed of single cells, small tumor nests, or a trabecular arrangement of tumor cells.

#### Intervascular background coloration

Brownish epithelium between microvessels in a brownish area visualized by NBI-ME was defined as intervascular background coloration [16, 17].

### A prospective multicenter study on the predictive value of type B vessels for invasion depth of SESCC

From January to August 2011, we prospectively enrolled 211 consecutive patients with SESCC lesions scheduled for ER in five high-volume centers (Table 3). Prior to enrollment, we used pretreatment imaging (CT and/or MRI) to confirm no findings of lymph node metastasis or distant metastasis of SESCC among any of the 211 patients. The depth of tumor invasion of the all lesions was estimated prior to treatment using a magnifying endoscope (GIF-H260Z or GIF-Q240Z; Olympus Corporation, Tokyo, Japan) combined with NBI. The estimation of tumor invasion depth by magnifying endoscopy was made based upon type B vessels in the JES classification and recorded in case report form. The aim of this study was to evaluate the diagnostic values of type B vessels in the pretreatment estimation of invasion depth of SESCC.

**Table 3:** Participating high-volume centers

Participated institutions	Number of patients
Saku Central Hospital Advanced Care Center	31
Saitama Cancer Center	40
Osaka Medical Center for Cancer and Cardiovascular Diseases	71
Niigata University Medical and Dental Hospital	41
Sendai City Medical Center	28

Informed consent was obtained from all participants. The informed consent includes information about the possible risks, benefits, and limits of the endoscopic procedure and treatment. We provided all patients with information about the possibility that additional treatments such as surgery or chemoradiotherapy will be required particularly if ER specimens would show SESCC invading the muscularis mucosa or deeper which is associated with increased risks of lymph node metastasis and distant metastasis. Especially for the patients with pretreatment NBI-ME diagnosis of B3 vessels, we provided the patients with sufficient explanation as follows: (1) B3 vessels will be a suggestive finding of tumor invasion of T1b-SM2 or deeper, (2) T1b-SM2 tumor are basically contraindication for ER because of considerably high risk of lymph node metastasis and distant metastasis, (3) additional therapy such as esophagectomy and chemoradiotherapy will be required if histology from ER would show tumor depth of T1b-SM2 or deeper.

We performed ER on all enrolled patients. The lesions were removed by endoscopic mucosal resection or endoscopic submucosal dissection. Using the resected specimens, the histological tumor invasion depth was established by pathologists in each center with expertise in gastrointestinal cancer, according to the Japanese classification of esophageal cancer [4]. None of the pathologists were aware of any endoscopic findings. We evaluated the diagnostic utility of the JES classification in predicting histological depth of invasion by comparing the pretreatment NBI-ME diagnosis with the histology from resected specimens.

## Results

All of the SESCC tumors enrolled in this study had type B vessels. The numbers of SESCC tumors subclassified as T1a-EP or T1a-LPM, T1a-MM or T1b-SM1, and T1b-SM2 were 163, 28, and 20, respectively.

The relationships between histology and pretreatment NBI-ME diagnosis of tumor invasion depth are listed in Table 4. One hundred fifty-nine (97.5 %) of the 163 T1a-EP or T1a-LPM tumors were accurately diagnosed by pretreatment NBI-ME, and four (2.5 %) were overestimated as T1a-MM or T1b-SM1. Twenty-one of 28 (75 %) T1a-MM or T1b-SM1 tumors were accurately diagnosed, and seven (25 %) were underestimated as T1a-EP or T1a-LPM. None of the T1a-EP or T1a-LPM/T1a-MM or T1b-SM1 tumors were overestimated as T1b-SM2. Eleven of 20 (55 %) T1b-SM2 tumors were accurately diagnosed. Six (30 %) and three (15 %) were underestimated as T1a-EP or T1a-LPM and T1a-MM or T1b-SM1, respectively. The overall accuracy of NBI-ME based upon the JES classification was 90.5 % (95 % confidence interval, 85.7–94.1 %).

**Table 4:** Relationships between histology and pretreatment ME-NBI diagnosis of depth of invasion of superficial squamous cell carcinomas

NBI-ME diagnosis	Histological invasion depth, *n*		
	T1a-EP or T1a-LPM	T1a-MM or T1b-SM1	T1b-SM2
B1 (T1a-EP or T1a-LPM)	159	7	6
B2 (T1a-MM or T1b-SM1)	4	21	3
B3 (T1b-SM2)	0	0	11

*NBI-ME* Narrow Band Imaging magnifying endoscopy, *EP* epithelium, *LPM* lamina propria mucosae, *MM* muscularis mucosae, *SM* submucosa, *n* Number of lesions

Table 5 shows the diagnostic values of B1, B2, and B3 vessels for estimating the depth of invasion of T1a-EP or T1a-LPM, T1a-MM or T1b-SM1, and T1b-SM2 tumors, respectively. The sensitivity/specificity/positive predictive value (PPV) and negative predictive value (NPV) of B1, B2, and B3 vessels were 97.5 %/72.9 %/92.4 % and 89.7 %; 75.0 %/96.2 %/75.0 % and 96.2 %; and 55 %/100 %/100 % and 95.5 %, respectively.

**Table 5:** Diagnostic values of type B vessels for estimating invasion depth of superficial squamous cell carcinomas

NBI-ME diagnosis	Sensitivity (95 % CI)	Specificity (95 % CI)	PPV (95 % CI)	NPV (95 % CI)	Accuracy (95 % CI)
B1 (T1a-EP or T1a-LPM)	97.5 (93.8–99.3)	72.9 (58.2–84.7)	92.4 (87.4–95.9)	89.7 (75.8–97.1)	91.9 (87.4–95.2)
B2 (T1a-MM or T1b-SM1)	75.0 (55.1–89.3)	96.2 (92.3–98.4)	75.0 (55.1–89.3)	96.2 (92.3–98.4)	93.4 (89.1–96.3)
B3 (T1b-SM2)	55.0 (31.5–76.9)	100 (98.1–100)	100 (71.5–100)	95.5 (91.6–97.9)	95.9 (92.1–98.0)

*NBI-ME* Narrow Band Imaging magnifying endoscopy, *EP* epithelium, *LPM* lamina propria mucosae, *MM* muscularis mucosae, *SM* submucosa, *CI* confidence interval, *PPV* positive predictive value, *NPV* negative predictive value

## Discussion

Endoscopic estimation of invasion depth is a key factor in deciding the treatment policy of SESCCs. We, the JES committee members, have developed a simplified magnifying endoscopic diagnosis classification (i.e., the JES classification). We conducted a prospective multicenter study focused on evaluating the diagnostic values of type B vessels in the classification for predicting the depth of invasion of SESCC.

The overall accuracy rate of the type B1, B2, and B3 microvessels was 90.5 %, which is of sufficiently high accuracy for clinical use. No T1a-EP or T1a-LPM or T1a-MM or T1b-SM1 tumor was overestimated as T1b-SM2. Although 45 % (9/20) of SM2 tumors did not have B3 vessels, B3 vessels may be highly suggestive of T1b-SM2 tumors because the PPV of B3 vessels was 100 %.

The sensitivity and PPV of B1 vessels for T1a-EP or T1a-LPM tumors were 97.5 and 92.4 %, respectively. Thus, B1 vessels will provide optimal decision-making regarding the indication for ER. In contrast, the sensitivity and PPV of B2 vessels for T1a-MM or T1b-SM1 tumors were both a suboptimal 75.0 %, while the diagnostic accuracy of white light endoscopy showed a lower value at 66–74 % [18]. Previous studies have indicated that T1a-MM or T1b-SM1 tumors without lymphovascular invasion had a significantly lower risk of lymph node metastasis and a good prognosis [19–21]. Therefore, T1a-MM or T1b-SM1 tumors are potential candidates for curative treatment by ER. Esophagectomy involves severe problems related to its high mortality rate and a decrease in quality of life after surgery [22]. Further study on the definition of B2 vessels is warranted to reduce the risk of unnecessary surgery for patients with T1a-MM or T1b-SM1 tumors.

In the JES classification, we partially modified definitions of AVA, which will be useful to predict invasion depth of SESCC as previous studies showed [11, 12]. Although AVA was not assessed in this study, we intend to investigate the predictive values of AVA and whether evaluation of AVA has a positive effect on the predictive value based upon type B vessels (especially for B2 vessels) in an upcoming study.

Arima et al. reported that reticular pattern (Type R) vessels were characteristic vascular structure reflecting a histologic infiltrative growth (i.e., INFc) of invasive SCC tumors or non-SCC types of malignant epithelial neoplasms. This study did not included non-SCC types of malignant epithelial neoplasms and the type R vessels were not seen in invasive SCC tumors included in this study. A validation study on type R vessels in predicting the histologic features of SCC and non-SCC tumors is warranted.

Intervascular background coloration will be useful for predicting histology of HGIN and invasive SCC [16, 17]. A recent study indicated that intervascular background coloration might be caused by an extravascular component of hemoglobin that is produced within tumor cells [23]. The other study showed that intervascular background coloration might be related to the thinning of the keratinous layer or the epithelium caused by neoplastic cell proliferation [17]. In this study, we did not evaluated diagnostic value of intervascular background coloration to predict HGIN and invasive SCC. The diagnostic validity and reliability of intervascular background coloration should be assessed in further multicenter studies.

This study had several limitations. First, the number of T1a-MM or T1b-SM1 and T1b-SM2 tumors was relatively small and no tumor resected by surgery was included. Further study including greater or adequate numbers of these tumors should be conducted to validate the results of the current study. Second, a central review on both the magnifying endoscopic findings and the histology was not conducted in this study. The endoscopic diagnosis and histology were left to each endoscopist and each pathologist in their respective hospitals. Thus, this study may lack diagnostic standardization in magnifying endoscopy and histology. Third, the endoscopists involved in this study are all experts. Additional study involving non-experts is needed to determine whether the results of this study on diagnostic values are applicable in general. Fourth, the reliability or reproducibility of the JES classification was not assessed in this study. It would be desirable to investigate inter- or intra-observer agreements in future studies.

## Conclusions

The newly developed JES classification is proposed to be useful in estimating invasion depth of SESCCs. Modification of the definition of B2 vessels will be necessary, and further studies are required to validate these study results as well as to improve the diagnostic utility of the JES classification.
